# Predictors of Prolonged Cardiopulmonary Exercise Impairment After COVID-19 Infection: A Prospective Observational Study

**DOI:** 10.3389/fmed.2021.773788

**Published:** 2021-12-24

**Authors:** Karin Vonbank, Antje Lehmann, Dominik Bernitzky, Maximilian Robert Gysan, Stefan Simon, Andrea Schrott, Martin Burtscher, Marco Idzko, Daniela Gompelmann

**Affiliations:** ^1^Department of Pulmonary Medicine, Medical University of Vienna, Vienna, Austria; ^2^StatistikAmbulanz, Leobendorf, Austria; ^3^Department of Sport Science, University of Innsbruck, Innsbruck, Austria

**Keywords:** COVID-19, exercise limitation, oxygen uptake, CPET, exercise capacity

## Abstract

**Objectives:** Coronavirus disease 2019 (COVID-19) is a global pandemic affecting individuals to varying degrees. There is emerging evidence that even patients with mild symptoms will suffer from prolonged physical impairment.

**Methods:** In this prospective observational study, lung function, and cardiopulmonary exercise testing have been performed in 100 patients for 3–6 months after COVID-19 diagnosis (post-CoVG). Depending on the severity of severe acute respiratory syndrome coronavirus type 2 (SARS-CoV-2) infection, patients were divided into asymptomatic, or mild to moderate (mild post-CoVG), and severe post-CoVG [hospitalization with or without intensive care unit/non-invasive ventilation (ICU/NIV)]. Results have been compared with age, sex, and body mass index (BMI) matched control group (CG, *N* = 50).

**Results:** Both lung function (resting) and exercise capacity (peak workload, Wpeak and peak oxygen uptake, VO_2_ peak - % predicted) were considerably affected in patients with severe post-CoV (81.7 ± 27.6 and 86.1 ± 20.6%), compared to the mild post-CoVG (104.8 ± 24.0%, *p* = 0.001 and 100.4 ± 24.8; *p* = 0.003). In addition, also the submaximal exercise performance was significantly reduced in the severe post-CoVG (predicted VT1/VO_2_ peak; *p* = 0.013 and VT2/VO_2_ peak; *p* = 0.001). Multiple linear regression analyses revealed that 74 % (adjusted *R*^2^) of the variance in relative VO_2_ peak of patients who had CoV could be explained by the following variables: lower age, male sex, lower BMI, higher DLCO, higher predicted heart rate (HR) peak, lower breathing reserve (BR), and lower SaO_2_ peak, which were related to higher relative VO_2_ peak values. Higher NT-proBNP and lower creatinine kinase (CK) values were seen in severe cases compared to patients who experienced mild CoV.

**Discussion:** Maximal and submaximal exercise performance in patients recovering from severe COVID-19 remain negatively affected for 3–6 months after COVID-19 diagnosis. The presented findings reveal that impaired pulmonary, cardiac, and skeletal muscle function contributed to the limitation of VO_2_ peak in those patients, which may have important implications on rehabilitation programs.

## Introduction

Coronavirus disease 2019 (COVID-19) is a global pandemic affecting individuals to varying degrees, ranging from few days of mild symptoms to respiratory distress requiring intensive care unit (ICU) treatment, including ventilation support and death (1, 2). Most acute respiratory syndrome coronavirus type 2 (SARS-CoV-2) infections are mild to moderate (3–5), but pneumonia progressing to acute respiratory failure is associated with significant morbidity and even mortality (6). The use of a minimum set of common outcome measures for COVID-19 studies (measurements of viral burden of patient survival and of patient progression) have been recommended by the International Forum for Acute Care Trialists, and the International Severe Acute Respiratory and Emerging Infections Consortium (7). It has been suggested that even patients with mild symptoms can suffer from prolonged physical impairment and reduced quality of life (QoL) (8). Although COVID-19 is a novel disease, there have been previous outbreaks of SARS-CoV, where deficits in cardiorespiratory and musculoskeletal performance (e.g., in the 6-min walking test, 6-MWT, and/or handheld dynamometry for major muscle groups), but also a reduced QoL was demonstrated in a cohort study of 171 SARS survivors (9). Besides the pulmonary involvement (10), there are increasing reports of non-pulmonary manifestations in patients with COVID-19, such as musculoskeletal abnormalities with pain and weakness in lower limbs (11–13), elevated creatinine kinase (CK), and lactate dehydrogenase (LDH) (12). Respiratory muscle's weakness and with higher ventilatory demands were reported even in patients recovering from mild COVID-19 symptoms (14). Moreover, cardiac complications like myocarditis may be the consequence of direct viral injury or cardiac damage due to the host's immune response (13, 15). These findings let us hypothesize that the majority of the patients with COVID-19 will present reduced exercise capacity and abnormal responses to cardiopulmonary exercise testing (CPET), even a few months after disease onset. Thus, the aims of this prospective study were (1) to assess exercise capacity and cardiopulmonary responses to CPET, and (2) to evaluate potentially limiting factors of aerobic exercise capacity in adult patients who had COVID-19 for 3–6 months after confirmation of infection.

## Methods

### Subjects

A total of 100 adult patients were enrolled in this prospective trial (between April 2020 and February 2021) at the Department of Pulmonology of the Medical University of Vienna. Included patients were those who recovered from asymptomatic or symptomatic COVID-19, confirmed by a positive reverse transcriptase PCR SARS-CoV-2 test obtained from a nasopharyngeal or oropharyngeal swab, and formed the post-COVID group (post-CoVG). For comparison purposes, an age/sex/body mass index (BMI)-matched control group (CG, *n* = 50) was established from a collective history of healthy individuals at the cardiorespiratory laboratory (tested between 2018 and 2020).

After clinical routine examinations, patients were subjected to lung function testing, blood tests with determination of NT – pro brain natriuretic peptide (NT-proBNP) concentration, as well as CK activity, symptom-limited CPET with analysis of gas exchange, blood gases, and lactate kinetics. Depending on the severity of SARS-CoV2 infection, patients were divided into two groups: asymptomatic or mild to moderate (mild post-CoVG) and severe post-CoVG [hospitalization with or without ICU/non-invasive ventilation (NIV)].

The study protocol was approved by the Ethics committee of the Medical University of Vienna (EC-1551/2020). All patients signed a written informed consent at the time of enrolment.

### Pulmonary Lung Function Test

Lung function was assessed by the use of body plethysmography and spirometry.

Predicted normal values were derived from the reference values in accordance with current recommendations (16). The following resting pulmonary function parameters were determined: vital and forced vital capacity (VC and FVC), forced expiratory volume in one second (FEV1), FEV1/FVC, total lung capacity (TLC), and residual volume (RV). Diffusing capacity for carbon monoxide (DLCO) and the CO transfer factor (KCO, DLCO/VA) were measured by single-breath technique. Results were expressed as absolute values and as percent of predicted values. Each value represents the best of at least three measurements. Spirometry and whole-body plethysmography, including DLCO, were performed with the Master Screen Body (FA Reiner/Viasys, Carefusion, Austria).

### Cardiopulmonary Exercise Test

All participants underwent a symptom-limited CPET on an Ergoline 800 bicycle (Sensormedics, United States) with respiratory gas-exchange analysis, using a step protocol with progressive increase in workload every minute according to a total exercise time between 8 and 12 min. The increment was adapted to the expected maximum working capacity. Patients were encouraged to exercise until exhaustion. A cycling frequency of 60–80 revolutions per minute (rpm) had to be maintained. Patients were encouraged to exercise until exhaustion. The test was stopped when the subject failed to maintain a pedal frequency of at least 60 rpm. Blood pressure was measured every 2 min and a continuous 12-lead electrocardiogram and peripheral oxygen saturation (SpO_2_) were recorded. Breath-by-breath minute ventilation (VE), carbon dioxide output (VCO_2_), and oxygen uptake (VO_2_) were measured using the Sensormedics 2900 Metabolic Measurement Cart (Carefusion GmbH, Höchberg, Germany). The respiratory exchange ratio (RER) was defined as VCO_2_/VO_2_, the oxygen pulse was calculated by VO_2_/heart rate, the ventilatory equivalent for oxygen uptake (VE/VO_2_), and the ventilatory equivalent for carbon dioxide production (VE/VCO_2_) were calculated. The VT1 was determined using the V-slope method, double-checked by establishing the nadir of VE/VO_2_ vs. work rate relationship. On the other hand, VT2 was determined using the point of increase of the VE vs. VCO_2_, double-checked by establishing the nadir of VE/VCO_2_ versus work rate relationship. Blood lactate (La) was measured at rest and every 2 min, at maximum exercise, as well as 1, 3, and 5 min of recovery time (BIOSEN-S-line, EKF diagnostic Leupamed, Austria). Blood gas analysis was performed at rest, at VT1, and at peak exercise (ABL800 Flex, Drott Medizintechnik GmbH, Austria). Maximum voluntary ventilation (MVV) was calculated as FEV1 multiplied by 35. Breathing reserve (BR) was calculated as VEpeak/MVV^*^100.

### Statistical Analysis

Statistical analyses were performed by IBM SPSS version 26.0 (IBM SPSS Statistics for Windows, Chicago, IL, USA). Normal distribution of data was verified by the Kolmogorov-Smirnov test and also by Shapiro-Wilk test. After a descriptive data analysis (number of cases and percent for categorial data and mean, SD, and range for continuous data), between CG and post-CoVG group differences in baseline characteristics, as well as between mild and severe post-CoVG, were analyzed using Student *t*-tests for normally distributed data, with homogeneous or heterogeneous variances; Mann-Whitney U-test was used for abnormally distributed data and for categorical data, Fisher's exact test was used for 2 x 2-crosstabs; Pearson's *X*^2^-test for larger crosstabs. Group differences between CG, mild and severe post-CoVG, were tested via analysis of variances (ANOVA) whenever normal distribution and homogeneity of variances and covariances were given, in case of significant group effect followed by Games-Howell *post-hoc* tests (because of unequal sample size and unequal standard deviation). For all the data where necessary conditions for ANOVA were not met, Kruskal-Wallis-Tests were calculated, in case of a significant group effect followed by pairwise Mann-Whitney U-tests (α-corrected by Bonferroni-Holm). Multiple linear regression (backward) was applied for VO_2_ peak (ml/kg/min) as dependent variable, using age, male sex, BMI, FEV1 (%), DLCO (%), Hrpeak (% of predicted, Tanaka), BR %, peak SpO_2_ (%), and % change of O_2_ pulse from VT1 to VO_2_ peak as potential predictors as those parameters showed significant group differences in previous univariate analyses. A two-sided level of significance of *p* = 0.05 was set.

## Results

### Baseline Characteristics

Baseline characteristics of the post-CoVG and the CG are shown in Table 1. The total study cohort consisted of 47.3% female and 52.7 % male individuals. The mean age of the total cohort was 46.8 (13.2) years; 46.4 (13.7) years in the CG, and 47 (13) in the post-CoVG. No significant differences were found between those groups regarding mean age, age group distribution, mean weight, mean BMI, and the frequency of comorbidities. More patients of the post-CoVG have reported allergies compared to the CG (26 vs. 10%, *p* = 0.031), whereas more active smokers were found in the CG (20 vs. 9%, *p* = 0.038). No significant differences have been detected concerning medication use, except for angiotensin converting enzyme (ACE) blockers (more frequent in the post-CoVG; 9 vs. 0%, *p* = 0.030). The median time from the diagnosis of COVID-19, as defined by positive SARS-CoV-2PCR testing, was 16 weeks.

**Table 1:** Baseline characteristics.

	**Total sample**	**CG**	**PostCG**	***p***
Participants, *n*	150	50	100	
Age (yrs), mean (SD) [range]	46.8 (13.2) [18–69]	46.4 (13.7) [18–68]	47.0 (13.0) [19–69]	0.797^#^
Age group (yrs), *n* (%)				
20– <30	21 (14.0)	8 (16.0)	13 (13.0)	0.993^###^
30– <40	22 (14.7)	6 (12.0)	16 (16.0)	
40– <50	40 (26.7)	14 (28.0)	26 (26.0)	
50– <60	39 (26.0)	12 (24.0)	27 (27.0)	
60– <70	28 (18.7)	10 (20.0)	18 (18.0)	
Height (cm), mean (SD) [range]	173.1 (9.6) [149–203]	170.9 (8.3) [157–188]	174.3 (10.0) [149–203]	*0.044^#^*
Weight (kg), mean (SD) [range]	80.3 (18.5) [44–149]	76.3 (15.7) [44–113]	82.4 (19.6) [50–149]	0.056^#^
BMI, mean (SD) [range]	26.7 (5.3) [17.0–26.7]	26.0 (4.4) [17.0–37.4]	27.0 (5.7) [17.0–37.4]	0.251^#^
Female sex, *n* (%)	71 (47.3)	27 (54.0)	44 (44.0)	0.299^§^
Smoking status, *n* (%)				
Non-smoker	91 (60.7)	32 (64.0)	59 (59.0)	*0.038^§§^*
Active smoker	19 (12.7)	10 (20.0)	9 (9.0)	
Former smoker	40 (26.7)	8 (16.0)	32 (32.0)	
Comorbidities, *n* (%)				
Cardiovascular disease	9 (6.0)	5 (10.0)	4 (4.0)	0.161^§^
Heart arrythmia	8 (5.3)	3 (6.0)	5 (5.0)	0.797^§^
Asthma	11 (7.3)	5 (10.0)	6 (6.0)	0.507^§^
Impaired LVF (left ventricular function)	1 (0.7)	0 (0.0)	1 (1.0)	0.478^§^
Diabetes	15 (10.0)	2 (4.0)	13 (13.0)	0.146^§^
Hypertension	32 (21.3)	10 (20.0)	22 (22.0)	0.835^§^
Chronic kidney disease	1 (0.7)	0 (0.0)	1 (1.0)	0.999^§^
Allergies	31 (20.7)	5 (10.0)	26 (26.0)	*0.031^§^*
Medication				
Aspirin	10 (6.7)	3 (6.0)	7 (7.0)	0.999^§^
Anticoagulants	4 (2.7)	1 (2.0)	3 (3.0)	0.999^§^
ACE blocker	9 (6.0)	0 (0.0)	9 (9.0)	*0.030^§^*
ATII blocker	14 (9.3)	7 (14.0)	7 (7.0)	0.232^§^
Betablocker	24 (16.0)	7 (14.0)	17 (17.0)	0.814^§^
Statine	23 (15.3)	11 (22.0)	12 (12.0)	0.148^§^
Immunosuppressive	3 (2.0)	0 (0.0)	3 (3.0)	0.551^§^
Arrhythmogenic	1 (0.7)	0 (0.0)	1 (1.0)	0.999^§^
ICS/LABA	13 (8.7)	5 (10.0)	8 (8.0)	0.761^§^
Thyrex	8 (5.3)	0 (0.0)	8 (8.0)	0.052^§^
Diuretica	4 (2.7)	0 (0.0)	4 (4.0)	0.302^§^
Calcium antagonist	7 (4.7)	2 (4.0)	5 (5.0)	0.999^§^

*CG, Control Group; PostCG, Post Covid Group; n, sample size (valid cases); yrs, years; SD, standard deviation*.

# *T-test for independent samples and homogeneous variances, two-sided*.

### *Mann-Whitney-U-test, two-sided*.

§ *Fisher's exact test for crosstabs, two-sided*.

§§ *Pearson's X^2^-test for crosstabs, two-sided*.

Lung function parameters of the post-CoVG (separate parameters for mild and severe disease) are shown in Table 2. Manifestation of COVID-19 at the time of the disease was asymptomatic at 3%, mild to moderate without hospitalization at 76%, patients needing hospital admission without non-invasive ventilation/ICU or intensive unit at 18%, and needing ICU or intensive unit at 3%. Static and dynamic lung volumes were significantly reduced in the hospitalized patients. Impaired diffusion capacity as defined by DLCO < lower limit of normal (LLN) was found in 65% of hospitalized patients, and in 29.5% in patients with mild post-CoV (*p* = 0.004). Blood gases at rest did not significantly differ between the two patient groups. Higher NT-proBNP and lower CK values were seen in severe compared to patients with mild post-CoV. No significant differences between both groups could be found concerning comorbidities except a higher percentage of diabetes in the patients with severe post-CoV.

**Table 2:** Post coronavirus disease 2019 (COVID-19) group by course of disease.

	**Total postCG**	**Mild postCG**	**Severe postCG**	***p***
Participants, n	100	79	21	
**COVID-19 manifestation**, ***n*** **(%)**				
Asymptomatic	3 (3.0)	3 (3.8)	NS	
Mild to moderate	76 (76.0)	76 (96.2)	NS	
Hospital admission	18 (18.0)	NS	18 (85.7)	
ICU/NIV	3 (3.0)	NS	3 (14.3)	
Diffusion impairment at exercise, *n* (%) {valid}	9 (9.5) {95}	5 (6.8) {74}	4 (19.0)	0.105^§^
Cardiac arrythmia at exercise, *n* (%) {valid}	28 (28.3) {99}	23 (29.1)	5 (25.0) {21}	0.475^§^
**Lung function**				
TLC (%), mean (SD)	100.6 (17.8)	103.2 (17.1)	91.1 (17.3)	*0.003^###^*
RV (%), mean (SD)	33.2 (7.4)	32.4 (7.1)	36.3 (7.8)	*0.029^###^*
FVC (%), mean (SD)	93.3 (16.6)	95.9 (13.8)	83.6 (22.3)	*0.025^##^*
FEV1 (l), mean (SD)	3.44 (0.97)	3.61 (0.97)	2.80 (0.68)	* <0.001^#^*
FEV1 (%), mean (SD)	94.2 (16.8)	96.5 (13.8)	85.6 (22.3)	*0.026^###^*
FEV1/FVC (%), mean (SD)	80.2 (5.5)	80.1 (5.1)	80.8 (7.0)	0.352^###^
DLCO (%), mean (SD) {valid}	82.9 (16.0) {98}	85.0 (14.8) {78}	74.8 (18.2) {20}	*0.010^#^*
DLCO < LLN, *n* (%) {valid}	36 (36.7) {98}	23 (29.5) {78}	13 (65.0) {20}	*0.004^§^*
KCO (%)	93.0 (12.2) {98}	93.4 (12.6) {78}	91.7 (10.9) {20}	0.576^#^
KCO < LLN, *n* (%) {valid}	2 (2.0) {98}	2 (2.6) {78}	0 (0.0) {20}	0.632^§^
**Blood gases at rest**				
paO_2_ (mmHg), mean (SD)	89.4 (9.8)	89.7 (9.7)	88.4 (10.5)	0.592^#^
paCO_2_ (mmHg), mean (SD)	35.3 (3.7)	35.1 (3.6)	36.0 (3.9)	0.334^#^
AaDO_2_ (mmHg), mean (SD)	17.5 (8.8)	17.4 (8.7)	17.9 (9.3)	0.591^###^
VECO_2_ (mmHg), mean (SD) {valid}	33.7 (5.3) {99}	33.6 (4.8) {78}	34.3 (6.9)	0.745^###^
VEO_2_ (mmHg), mean (SD) {valid}	27.7 (5.0) {99}	27.5 (4.6) {78}	28.4 (6.4)	0.857^###^
proBNP, mean (SD) {valid}	63.4 (91.1) {97}	52.7 (71.5) {76}	134.8 (83.1)	0.005^###^
CK, U/l, mean (SD) {valid}	125.9 (78.9) {96}	101.9 (136.5) {75}	94.3 (51.4)	0.016^###^

*PostCG, Post Covid Group; n, sample size (valid cases); SD, standard deviation*.

# *T-test for independent samples and homogeneous variances, two-sided*.

## *T-test for independent samples and heterogeneous variances, two-sided*.

### *Mann-Whitney-U-test, two-sided*.

§ *Fisher's exact test for crosstabs, two-side*.

### CPET Parameters of the Post-CoVG and CG

**Figure 1:**
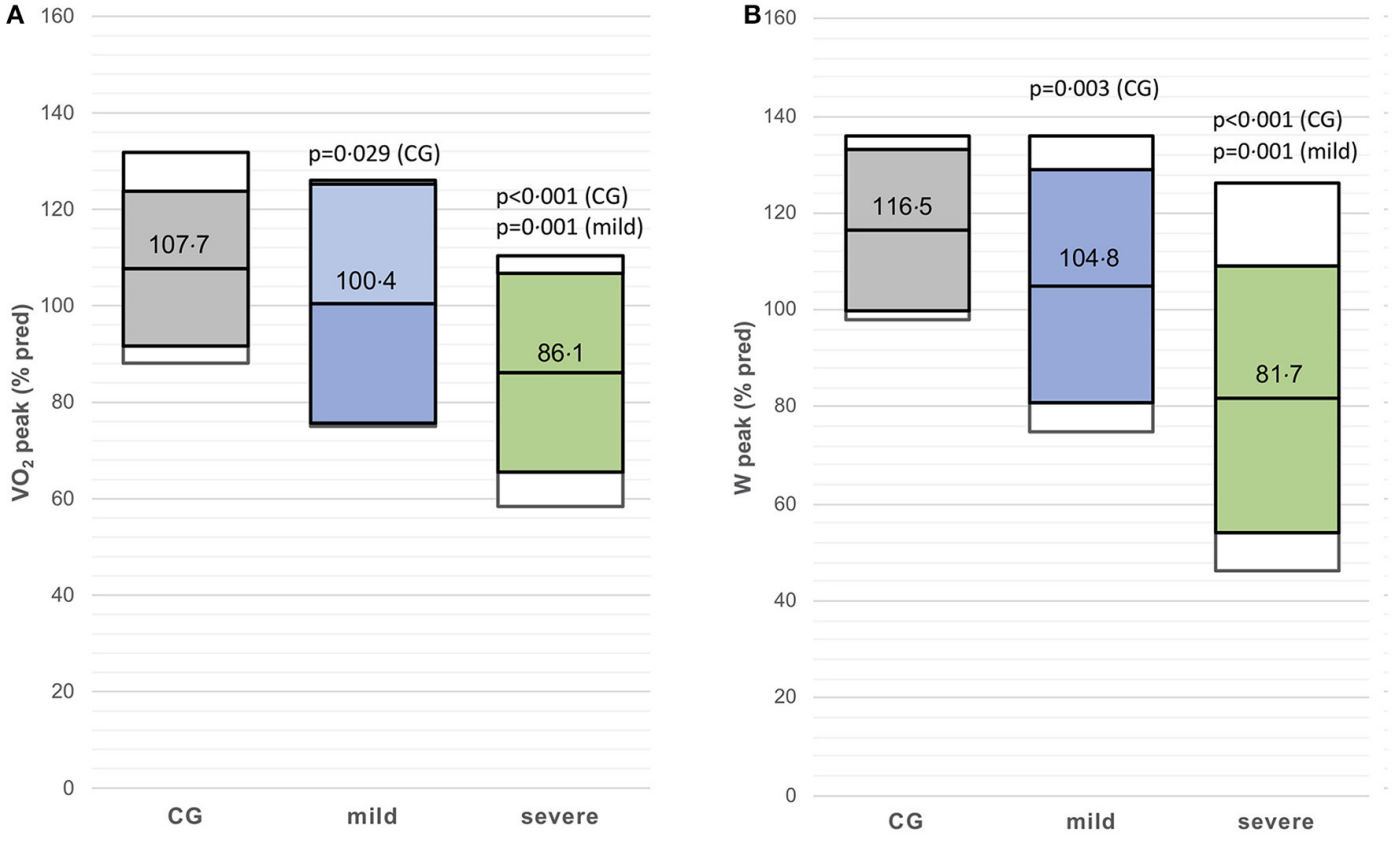
**(A,B)** VO_2_peak (% predicted) and Wpeak (% predicted) in the control group (CG), mildly and severely affected post-coronavirus disease 2019 (COVID) patients. Colored boxes indicate mean ± SD, white boxes 90th percentiles.

**Figure 2:**
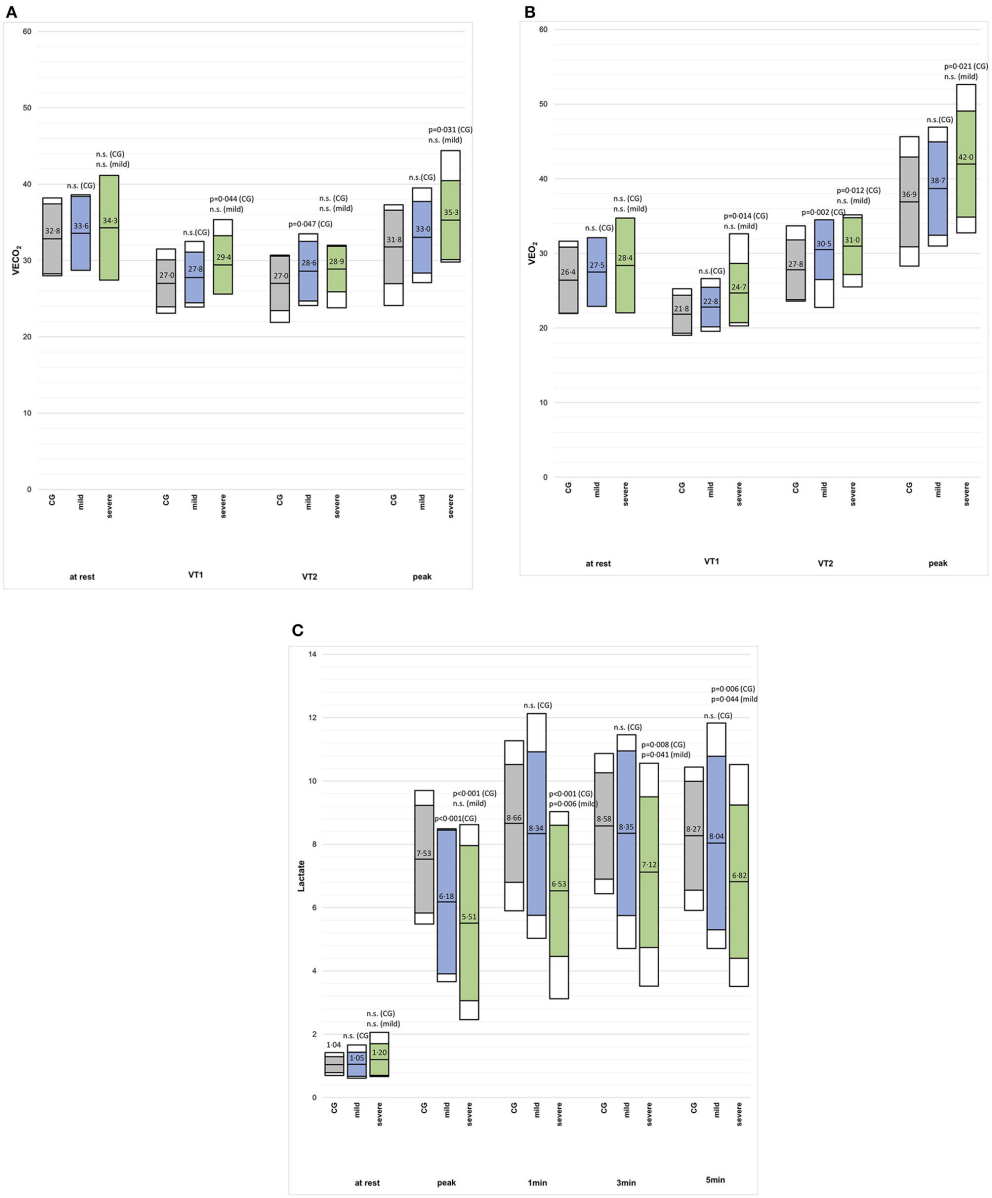
**(A–C)** VE/VCO_2_, VE/VO_2_ and blood lactate concentration at rest, VT1, VT2, and peak exercise, and the first 3 min post-exercise (2c) in in the control group (CG), mildly and severely affected post-COVID patients. Colored boxes indicate mean ± SD, white boxes 90th percentiles.

The VO_2_peak (%predicted) and Wpeak (%predicted) were lower in the mild post-CoVG compared to the CG (*p* = 0.003), the VO_2_peak (%predicted) and Wpeak (%predicted) were significantly lower in the severe post-CoVG compared to the other groups (CG vs. severe, *p* < 0.001, *p* = 0.001; mild vs. severe, *p* < 0.001, *p* = 0.003; Figures 1A,B). Differences of ventilatory equivalents at rest, VT1, VT_2_, and peak exercise between all three groups are depicted in Figures 2A,B. A significant higher VE/VO_2_ at VT2 and peak exercise could also be seen in mild post-COVG, compared to the control group as well as in the severe post-CoVG. In addition a significantly higher VE/VCO_2_ could also be found at VT1 and peak exercise in the post-CoVG compared to the CG. Lactate values at peak exercise were significantly lower in both patient groups compared to the CG (Figure 2C). In addition to those values, relative VO_2_peak, Hrpeak, VT1/VCO_2_, and VT1/VO_2_ predicted were all lower in the severe post-CoVG compared to the CG. All those values (plus HRs at VT1) were lower in the severe vs. the mild post-CoVG. No overall differences between groups were found for BR, VE at rest, and Vepeak (Table 3).

**Table 3:** Control group vs. study groups by course of disease—performance parameters.

	**CG**	**PostCG**		**p** **(3-groups)**	***Post-hoc*** **tests**		
		**Mild**	**Severe**		**CG vs. mild**	**CG vs. severe**	**Mild vs. severe**
Participants, *n*	50	79	21				
	**Mean (SD) {valid n}**	**Mean (SD) {valid n}**	**Mean (SD) {valid n}**				
W peak (%pred)	116.5 (16.5)	104.8 (24.0)	81.7 (27.6)	* <0.001^£££^*	*0.003*	* <0.001*	*0.001*
VO_2_ peak (ml/kg/min)	29.6 (7.5)	28.2 (9.0)	21.3 (6.4)	*0.001^£^*	0.613	* <0.001*	*0.001*
VO_2_ peak (%pred)	107.7 (16.0)	100.4 (24.8)	86.1 (20.6)	* <0.001^£££^*	*0.029*	* <0.001*	*0.003*
BR (%)	22.5 (19.7)	19.9 (15.1) {77}	17.1 (14.8)	0.441^£^	NS	NS	NS
Lactate peak (mmol/L)	7.53 (1.7)	6.18 (2.27)	5.51 (2.45)	* <0.001^£££^*	* <0.001*	* <0.001*	0.149
HR rest (bpm)	75.8 (11.5)	79.5 (11.3)	77.3 (9.7)	0.195^£^	NS	NS	NS
HR peak (bpm)	164.9 (18.9)	165.6 (22.1)	145.1 (25.7)	*0.001^£££^*	0.576	*0.001*	* <0.001*
VE rest (L)	10.9 (3.4)	11.7 (93.4) {78}	12.4 (3.2)	0.086^£££^	NS	NS	NS
VE peak (L)	86.6 (28.3)	93.4 (28.2)	78.1 (19.3)	0.058^£^	NS	NS	NS
VT1/VO_2_ pred (%)	55.0 (8.4) {48}	52.8 (9.9)	46.1 (9.7)	0.003^£££^	0.096	*0.001*	*0.013*
VT2/VO_2_ pred (%)	88.9 (13.7) {48}	89.0 (18.8) {78}	70.4 (18.2) {20}	* <0.001^£^*	0.999	*0.001*	*0.001*
HR at VT1 (bpm)	108.6 (16.1) {49}	110.1 (15.0)	100.7 (14.7)	*0.045^£^*	0.851	0.125	*0.036*
HR at VT2 (bpm)	142.4 (18.8) {49}	150.7 (23.0) {78}	126.1 (21.4) {20}	* <0.001^£££^*	*0.020*	*0.004*	* <0.001*

*CG, Control Group; PostCG, Post Covid Group; n, sample size (valid cases); %pred, percent predicted; bpm, beats per minute*.

£ *ANOVA (analysis of variances)*.

££ *Kruskal-Wallis-Tests (nonparametric, for >2 indepent samples, two-sided)*.

Multiple linear regression analyses (backward; *p* < 0.001) revealed that 74 % (adjusted *R*^2^) of the variance in relative VO_2_peak of patients with post-CoV could be explained by 7 variables (Table 4). Lower age, male sex, lower BMI, higher DLCO, higher predicted Hrpeak, lower BR, and lower SaO_2_peak are related to higher relative VO_2_peak values, while FEV1 and O_2_pulse (% change from VT1 to VO_2_peak) had no effect on VO_2_peak, and, thus, were excluded from the model.

**Table 4:** Results of the multiple linear regression backward (step 3).

	***p***	**B**	**95% CI for B**		**Standard error of B**
			**LL**	**UL**	
Total model	* <0.001*				
Age	* <0.001*	−0.251	−0.337	−0.164	0.044
Male sex	* <0.001*	4.552	2.543	6.561	1.011
BMI	* <0.001*	−0.682	−0.863	−0.501	0.091
DLCO (%)	* <0.001*	0.117	0.054	0.181	0.032
HRpeak (% of predicted)	* <0.001*	*0.189*	0.098	0.279	0.045
BR (%)	*0.009*	−0.095	−0.166	−0.025	0.035
SpO_2_ peak (%)	*0.045*	−0.114	−0.226	−0.002	0.056
(Constant)	* <0.001*	40.342	24.571	56.113	7.935
Adjusted R^2^	0.739				

*Dependent variable: VO_2_ peak (ml/kg/min). Independent variables included in step 1: age, male sex, BMI, FEV1 (%), DLCO (%), HRpeak (% of predicted, Tanaka), BR %, SpO_2_ (%) and O_2_ plus % change VT1 to max (sample size n = 98, postCG)*.

*CI, Confidence interval; B, Unstandardized Coefficient B; LL, 95% lower limit of confidence interval B; UL, 95% upper limit of confidence interval of B*.

## Discussion

This prospective study demonstrated prolonged impairment of both the predicted maximal exercise capacity (Wpeak and VO_2_peak) in patients with mild and severe post-CoV, and the predicted submaximal exercise performance (predicted VT1/VO_2_peak and VT2/VO_2_peak) only in patients with severe post-CoV compared to controls. Impairments were more pronounced in the severe compared to the mild post-CoVG. Regression analyses (supported by the elevated NT-proBNP values) revealed that aside from impaired pulmonary function, cardiac and skeletal muscle dysfunction contributed to the limitation of VO_2_peak in patients with post-CoV.

Generally, the manifestations of COVID-19 showed a wide severity spectrum from asymptomatic to most severe forms (17). Moreover, a large heterogeneity concerning the signs and the symptom duration was found in these patients (18–20). Not surprisingly, a reduced exercise capacity has been reported in 59% of 18 patients with COVID-19 at the time of hospital discharge, with a VO_2_peak <70% of predicted in 61% of cases (14). In agreement with our findings, prolonged performance impairment was demonstrated in 50% of patients with COVID history, up to 100 days after infection, with impaired lung function in 42% after 60 days, and still in 36% after 100 days (21). Another study reported the persistence of at least one symptom in more than 80% of patients with COVID-19 after the onset of the first symptoms (19). Diminished physical fitness associated with persistent breathing disorder has been found even 6 months after hospitalization for COVID-19 infection (22).

To our knowledge, we presented the first study demonstrating different impairment of maximal and submaximal exercise performance, between patients with mild and severe CoV history after 3–6 months of recovery. In addition, we showed that both impaired pulmonary and cardiac function, but also a probable dysfunction of the skeletal working muscles, contributed to the explanation of the reduced aerobic exercise capacity. Although VEmax and BR, as potential indicators for ventilatory limitation, were rather normal in the patients with CoV history, multiple linear regression analysis indicated that low BR contributed to the variance explanation of relative VO2max. This is also true for the compromised resting pulmonary lung function observed, which is in agreement with other studies (23, 24). Out of the patients with history in CoV, 37% showed decreased DLCO (< LLN), but without abnormal KCO (< LLN). These observations are also in accordance with previous studies (25, 26), supporting the view that loss of alveolar units is not the primary cause for the observed DLCO impairment (27). Thus, other factors like pulmonary microangiopathy, fibrin clotting within small capillaries around alveoli, small vessel thrombosis, and thickening of alveolar capillaries might contribute to a reduced DLCO (27).

The present findings suggest that aerobic exercise capacity (relative VO_2_peak) may also be limited as related to potential extrapulmonary manifestations, e.g., myopathy, autonomic dysregulation, and physical deconditioning (13, 15). Cardiac involvement is supported by the elevated NT-proBNP values in post-CoV patients. A correlation between left ventricular dysfunction, NT-proBNP, and exercise capacity has been demonstrated in patients with heart-failure (28). Cardiac abnormalities are common in survivors of COVID-19, and could be detected in 42.3% of the 97 survivors of the said illness (29), with a wide range of different manifestations including brady-tachyarrhythmia and COVID 19- related-myocarditis up to left ventricular dysfunction [12]. In our patients, cardiac arrythmia at exercise could be found in 28.3% of the patients who had CoV, without significant differences in both patient groups.

Whether it does and to what extent it can, the contribution of the dysfunction of working muscles to the observed VO2peak impairment is difficult to assess from CPET results. Severe muscle dysfunction and pain were reported in patients with COVID-19 (11, 12), and seemed also to be true in patients of the present study. This can be derived from the low HRmax values observed, likely indicating muscle dysfunction, as low maximal HR responses have also been demonstrated in patients suffering from chronic fatigue syndrome with debilitating fatigue, muscle pain, and muscle weakness (30). In addition, low VTs may also point to affected working muscles, which has been demonstrated in patients suffering from mitochondrial dysfunction (31).

There are several limitations of the current study. First, the study population consisted of a higher percentage of patients with history of COVID, with mild to moderate courses of the disease, but especially for these patients, information about cardiopulmonary limitations were missing. Secondly, cardiopulmonary evaluation prior to COVID-19 was not available, so it cannot fully evaluate the impact of pre-existing cardiopulmonary impairments. Although cardiorespiratory and musculoskeletal limitations have also been reported after previous outbreaks of SARS-CoV (9), it remains unclear that these findings are specific for COVID infections.

## Conclusions

Three to 6 months after recovery from COVID-19, many patients are still affected by reduced maximal exercise capacity (for mildly and severely affected patients) and by impaired submaximal exercise performance (only severely affected patients). Our findings reveal that pulmonary, cardiac and skeletal muscle dysfunction contributed to the limitations of aerobic exercise performance in patients with CoV history. These findings may have important implications on rehabilitation programs for the said patients, with a need for multidisciplinary collaborations to assess the optimal structure of rehabilitation programs.

## Data Availability Statement

The original contributions presented in the study are included in the article/supplementary material, further inquiries can be directed to the corresponding author/s.

## Ethics Statement

The studies involving human participants were reviewed and approved by the Ethics Committee of Medical University of Vienna. The patients/participants provided their written informed consent to participate in this study.

## Author Contributions

KV designed the study, had full access to all of the data used, acquired data, analyzed and interpreted data, searched literature, wrote, and reviewed and edited original draft. AL, DB, MG, and SS performed study examination and acquired data. AS and MB had full access to all data in the study, analyzed and interpreted data, wrote, and reviewed and edited original draft. MI and DG designed the study, had full access to all data in the study and verified the underlying data, and reviewed and edited original draft. All authors contributed to the article and approved the submitted version.

## Conflict of Interest

The authors declare that the research was conducted in the absence of any commercial or financial relationships that could be construed as a potential conflict of interest.

## Publisher's Note

All claims expressed in this article are solely those of the authors and do not necessarily represent those of their affiliated organizations, or those of the publisher, the editors and the reviewers. Any product that may be evaluated in this article, or claim that may be made by its manufacturer, is not guaranteed or endorsed by the publisher.

## Abbreviations

ACE: angiotensin converting enzyme
BMI: Body mass index
BR: Breathing reserve
CG: Control group
CK: Creatinine kinase
CPET: Cardiopulmonary exercise testing
DLCO: Diffusion capacity for carbon monoxide
FEV1: Forced expiratory volume in one second
HRpeak: Peak heart rate
ICU: Intensive care unit
KCO: carbon monoxide transfer factor
La: Blood lactate
LDH: lactate dehydrogenase
LLN: lower limit of normal
MVV: maximum minute ventilation
NIV: non-invasive ventilation
NT- pro BNP: Pro-brain natiuretic peptide
Post-CoVG: Post-COVID-infection group
QoL: quality of life
RER: Respiratory exchange ratio
RV: Residual volume
SARS-CoV-2: Severe acute respiratory syndrome coronavirus type 2
6-MWT: Six-minute walking test
TLC: Total lung capacity
VC: Vital capacity
VCO_2_: carbon dioxide output
VE: minute ventilation
VE/VO_2_: Ventilatory equivalent for oxygen uptake
VE/VCO_2_: Ventilatory equivalent for carbon dioxide production
VO_2_: Oxygen uptake
VT1: First ventilatory threshold
VT2: Second ventilatory threshold
Wpeak: Peak workload.
